# Supplementary material for: Distinct subpopulations of DN1 thymocytes exhibit preferential γδ T lineage potential
Source: Front Immunol. 2023 Apr 3;14:1106652. doi: 10.3389/fimmu.2023.1106652 (PMC10106834; doi:10.3389/fimmu.2023.1106652)
Supplement: Supplementary file 1 [file DataSheet_1.pdf]

SUPPLEMENTARY TABLE 1: Antibodies used for study

**Antibody**	**Clone**
Anti-mouse B220	RA3-6B2
Anti-mouse CD117	ACK2
Anti-mouse CD11b	M1/70
Anti-mouse CD11c	N418
Anti-mouse CD24	M1/69
Anti-mouse CD25	PC61.5
Anti-mouse CD317	eBio927
Anti-mouse CD3e	145-2C11
Anti-mouse CD4	RM4-5
Anti-mouse CD4	RM4-5
Anti-mouse CD44	IM7
Anti-mouse CD53	OX-79
Anti-mouse CD8α	53-6.7
Anti-mouse CD90.2	53-2.1
Anti-mouse IFNγ	XMG1.2
Anti-mouse IL-17A	eBio17B7
Anti-mouse Ly6d	49-H4
Anti-mouse Sca1	D7
Anti-mouse NK1.1	PK136
Anti-mouse NKG2D	CX5
Anti-mouse TCRβ	H57-597
Anti-mouse TCRγδ	eBioGL3
Anti-mouse TCRVγ1.1	2.11
Anti-mouse TCRVγ2	UC3-10A6

SUPPLEMENTARY TABLE 2. *P*-values comparing αβ versus γδ differentiation from DN1 subpopulations after 14d on OP9-DL1 monolayer (related to Figure 4B)

**Comparing % $CD8^+$**									
p-value	**DN1**	**1a**	**1b**	**1c**	**1d**	**1e-1**	**1e-2**	**1e-3**	**1e-4**
**DN1**		ns	****	****	****	****	****	****	****
**1a**	ns		****	****	****	****	****	****	****
**1b**	****	****		ns	****	****	****	****	****
**1c**	****	****	ns		****	****	****	****	****
**1d**	****	****	****	****		ns	ns	ns	ns
**1e-1**	****	****	****	****	ns		ns	ns	ns
**1e-2**	****	****	****	****	ns	ns		ns	ns
**1e-3**	****	****	****	****	ns	ns	ns		ns
**1e-4**	****	****	****	****	ns	ns	ns	ns	
**Comparing % TCRγδ$^+$**									
p-value	**DN1**	**1a**	**1b**	**1c**	**1d**	**1e-1**	**1e-2**	**1e-3**	**1e-4**
**DN1**		ns	ns	ns	ns	****	***	*	****
**1a**	ns		ns	ns	*	****	****	****	****
**1b**	ns	ns		ns	*	****	****	****	****
**1c**	ns	ns	ns		ns	****	****	****	****
**1d**	ns	*	*	ns		**	ns	ns	ns
**1e-1**	****	****	****	****	**		ns	*	ns
**1e-2**	****	****	****	****	ns	ns		ns	ns
**1e-3**	*	****	****	****	ns	*	ns		ns
**1e-4**	***	****	****	****	ns	ns	ns	ns	

The data was analyzed by one-way analysis of variance (ANOVA). ns = not significant, *$P<0.05$, ** $P<0.01$, *** $P<0.001$, **** $P<0.0001$.

SUPPLEMENTARY TABLE 3. *P*-values comparing αβ versus γδ differentiation from DN1 subpopulations after 20d on OP9-DL1 monolayer (related to Figure 4B)

**Comparing % CD8+**									
p-value	**DN1**	**1a**	**1b**	**1c**	**1d**	**1e-1**	**1e-2**	**1e-3**	**1e-4**
**DN1**		ns	ns	****	****	****	****	****	****
**1a**	ns		ns	****	****	****	****	****	****
**1b**	ns	ns		****	****	****	****	****	****
**1c**	****	****	****		****	****	****	****	****
**1d**	****	****	****	****		ns	ns	ns	ns
**1e-1**	****	****	****	****	ns		ns	ns	ns
**1e-2**	****	****	****	****	ns	ns		ns	ns
**1e-3**	****	****	****	****	ns	ns	ns		ns
**1e-4**	****	****	****	****	ns	ns	ns	ns	
**Comparing % TCRγδ+**									
p-value	**DN1**	**1a**	**1b**	**1c**	**1d**	**1e-1**	**1e-2**	**1e-3**	**1e-4**
**DN1**		ns	ns	ns	ns	****	****	*	***
**1a**	ns		ns	ns	**	****	****	***	****
**1b**	ns	ns		ns	***	****	****	****	****
**1c**	ns	ns	ns		ns	****	****	ns	*
**1d**	ns	**	***	ns		*	**	ns	ns
**1e-1**	****	****	****	****	*		ns	**	ns
**1e-2**	****	****	****	****	**	ns		***	*
**1e-3**	*	***	****	ns	ns	**	***		ns
**1e-4**	***	****	****	*	ns	ns	*	ns	

The data was analyzed by one-way analysis of variance (ANOVA). ns = not significant, * $P<0.05$, ** $P<0.01$, *** $P<0.001$, **** $P<0.0001$.

SUPPLEMENTARY TABLE 4. *P*-values comparing αβ versus γδ differentiation from DN2 subpopulations after 14d on OP9-DL1 monolayer (related to Figure 4D)

**Comparing % $CD8^+$**												
p-value	**DN2**	**2a-1**	**2a-2**	**2a-3**	**2a-4**	**2b-1**	**2b-2**	**2b-3**	**2b-4**	**2b-5**	**2b-6**	**2b-7**
**DN2**		*	****	***	****	ns	ns	***	ns	ns	ns	ns
**2a-1**	*		ns	ns	ns	****	ns	****	***	**	ns	****
**2a-2**	****	ns		ns	ns	****	***	****	****	****	ns	****
**2a-3**	***	ns	ns		ns	****	***	****	****	****	ns	****
**2a-4**	****	ns	ns	ns		****	****	****	****	****	ns	****
**2b-1**	ns	****	****	****	****		*	ns	ns	ns	****	ns
**2b-2**	ns	ns	***	***	****	*		****	ns	ns	ns	ns
**2b-3**	***	****	****	****	****	ns	****		*	***	****	ns
**2b-4**	ns	***	****	****	****	ns	ns	*		ns	**	ns
**2b-5**	ns	***	****	****	****	ns	ns	***	ns		*	ns
**2b-6**	ns	ns	ns	ns	ns	****	ns	****	**	*		***
**2b-7**	ns	****	****	****	****	ns	ns	ns	ns	ns	***	
**Comparing % TCRγδ^+**												
p-value	**DN2**	**2a-1**	**2a-2**	**2a-3**	**2a-4**	**2b-1**	**2b-2**	**2b-3**	**2b-4**	**2b-5**	**2b-6**	**2b-7**
**DN2**		ns	*	*	ns	ns	ns	ns	ns	ns	ns	ns
**2a-1**	ns		*	*	ns	ns	ns	ns	ns	ns	ns	ns
**2a-2**	*	*		ns	ns	**	*	***	ns	ns	ns	*
**2a-3**	*	*	ns		ns	*	ns	**	ns	ns	ns	ns
**2a-4**	ns	ns	ns	ns		ns	ns	*	ns	ns	ns	ns
**2b-1**	ns	ns	**	*	ns		ns	ns	ns	ns	ns	ns
**2b-2**	ns	ns	*	ns	ns	ns		ns	ns	ns	ns	ns
**2b-3**	ns	ns	***	**	*	ns	ns		ns	ns	ns	ns
**2b-4**	ns	ns	ns	ns	ns	ns	ns	ns		ns	ns	ns
**2b-5**	ns	ns	ns	ns	ns	ns	ns	ns	ns		ns	ns
**2b-6**	ns	ns	ns	ns	ns	ns	ns	ns	ns	ns		ns
**2b-7**	ns	ns	*	ns	ns	ns	ns	ns	ns	ns	ns	

The data was analyzed by one-way analysis of variance (ANOVA). ns = not significant, * $P<0.05$, ** $P<0.01$, *** $P<0.001$, **** $P<0.0001$.

SUPPLEMENTARY TABLE 5. *P*-values comparing αβ versus γδ differentiation from DN2 subpopulations after 20d on OP9-DL1 monolayer (related to Figure 4D)

**Comparing % $CD8^+$**												
p-value	**DN2**	**2a-1**	**2a-2**	**2a-3**	**2a-4**	**2b-1**	**2b-2**	**2b-3**	**2b-4**	**2b-5**	**2b-6**	**2b-7**
**DN2**		ns	ns	ns	ns	ns	ns	ns	*	*	ns	ns
**2a-1**	ns		ns	ns	ns	ns	ns	ns	**	**	ns	ns
**2a-2**	ns	ns		ns	ns	ns	ns	ns	ns	ns	ns	ns
**2a-3**	ns	ns	ns		ns	ns	ns	ns	ns	ns	ns	ns
**2a-4**	ns	ns	ns	ns		ns	ns	ns	ns	ns	ns	ns
**2b-1**	ns	ns	ns	ns	ns		ns	ns	**	**	ns	ns
**2b-2**	ns	ns	ns	ns	ns	ns		ns	ns	ns	ns	ns
**2b-3**	ns	ns	ns	ns	ns	ns	ns		ns	ns	ns	ns
**2b-4**	*	**	ns	ns	ns	**	ns	ns		ns	ns	**
**2b-5**	*	**	ns	ns	ns	**	ns	ns	ns		ns	***
**2b-6**	ns	ns	ns	ns	ns	ns	ns	ns	ns	ns		ns
**2b-7**	ns	ns	ns	ns	ns	ns	ns	ns	**	***	ns	
**Comparing % $TCR\gamma\delta^+$**												
p-value	**DN2**	**2a-1**	**2a-2**	**2a-3**	**2a-4**	**2b-1**	**2b-2**	**2b-3**	**2b-4**	**2b-5**	**2b-6**	**2b-7**
**DN2**		ns	ns	ns	ns	ns	ns	ns	ns	ns	ns	ns
**2a-1**	ns		**	ns	ns	ns	ns	ns	**	ns	ns	ns
**2a-2**	ns	**		**	ns	ns	ns	ns	ns	**	*	ns
**2a-3**	ns	ns	ns		ns	ns	ns	ns	**	ns	ns	ns
**2a-4**	ns	ns	ns	ns		ns	ns	ns	*	ns	ns	ns
**2b-1**	ns	ns	ns	ns	ns		ns	ns	ns	ns	ns	ns
**2b-2**	ns	ns	ns	ns	ns	ns		ns	*	ns	ns	ns
**2b-3**	ns	ns	ns	ns	ns	ns	ns		ns	ns	ns	ns
**2b-4**	ns	**	ns	**	*	ns	*	ns		**	*	ns
**2b-5**	ns	ns	**	ns	ns	ns	ns	ns	**		ns	ns
**2b-6**	ns	ns	*	ns	ns	ns	ns	ns	*	ns		ns
**2b-7**	ns	ns	ns	ns	ns	ns	ns	ns	ns	ns	ns	

The data was analyzed by one-way analysis of variance (ANOVA). ns = not significant, * $P<0.05$, ** $P<0.01$, *** $P<0.001$, **** $P<0.0001$.

SUPPLEMENTARY TABLE 6. *P*-values comparing Vγ1.1^{+} TCRγδ versus Vγ2^{+} TCRγδ cells produced from DN1 subpopulations after 14d on OP9-DL1 monolayer (related to Figure 4D)

**Comparing % Vγ1.1^{+} TCRγδ**							
p-value	**DN1**	**1c**	**1d**	**1e-1**	**1e-2**	**1e-3**	**1e-4**
**DN1**		ns	ns	****	***	ns	ns
**1c**	ns		*	****	****	ns	*
**1d**	ns	*		*	*	ns	ns
**1e-1**	****	****	*		ns	****	**
**1e-2**	***	****	*	ns		***	**
**1e-3**	ns	ns	ns	****	***		ns
**1e-4**	ns	*	ns	**	**	ns	
**Comparing % Vγ2^{+} TCRγδ**							
p-value	**DN1**	**1c**	**1d**	**1e-1**	**1e-2**	**1e-3**	**1e-4**
**DN1**		ns	ns	ns	ns	ns	ns
**1c**	ns		ns	ns	ns	ns	ns
**1d**	ns	ns		ns	ns	ns	ns
**1e-1**	ns	ns	ns		ns	ns	ns
**1e-2**	ns	ns	ns	ns		ns	ns
**1e-3**	ns	ns	ns	ns	ns		ns
**1e-4**	ns	ns	ns	ns	ns	ns	

The data was analyzed by one-way analysis of variance (ANOVA). ns = not significant, * $P<0.05$, ** $P<0.01$, *** $P<0.001$, **** $P<0.0001$.

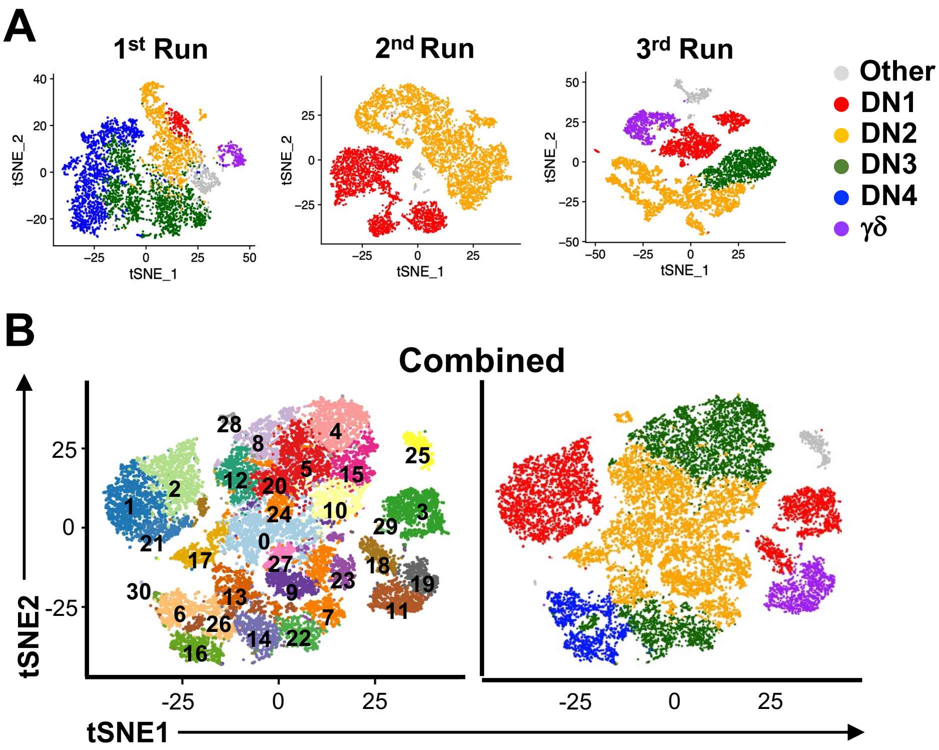

SUPPLEMENTARY FIGURE 1. t-SNE projection of the 10x scRNAseq data shown in Figure 1. **(A)** The three separate datasets were color-coded to DN developmental stage or TCRγδ$^+$ thymocytes or other (non-thymocytes). **(B)** The three datasets were integrated with SCTransform, then clustered with Seurat at at resolution of 2.0. The resulting clusters (left plot) were then annotated to DN developmental stage, TCRγδ$^+$ thymocytes or other (non-thymocytes) (right plot).

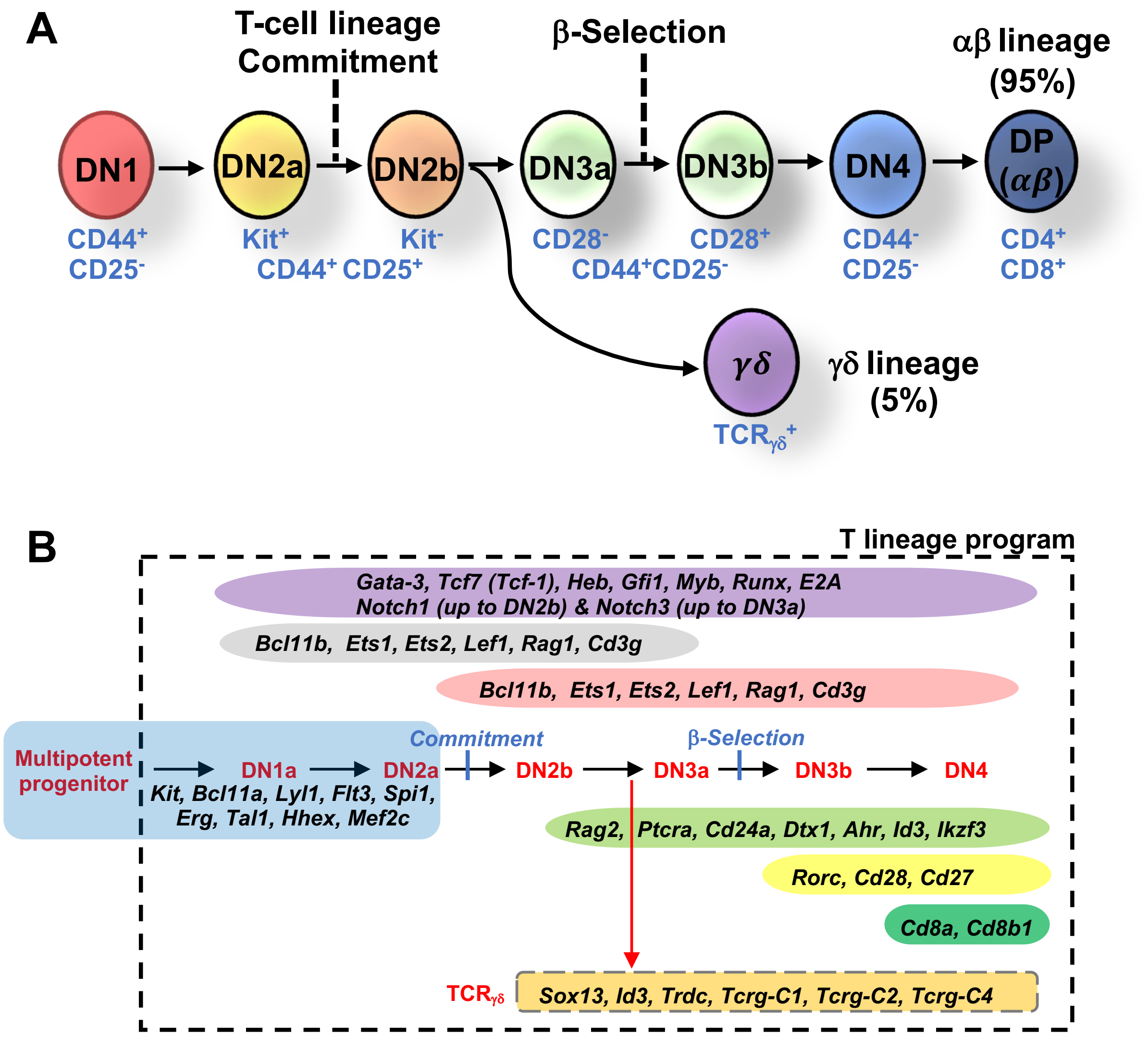

SUPPLEMENTARY FIGURE 2. The standard model of murine T cell development. **(A)** T cells are divided into two main lineages, αβ and γδ T cells, which are defined by T cell receptor (TCR) chain expression. Shown is a schematic overview of early T cell development, which is divided into four stages, termed double negative (DN) 1 to 4, based on expression of key cell surface markers. **(B)** The key cell surface markers are indicated. The current model assumes the γδ lineage branches off from αβ lineage at DN2b>DN3a, when *Tcrb/g/d* gene rearrangements occur. Also shown is a summary of the expression patterns of key marker genes that define the DN stages of T cell development as defined at a population level.

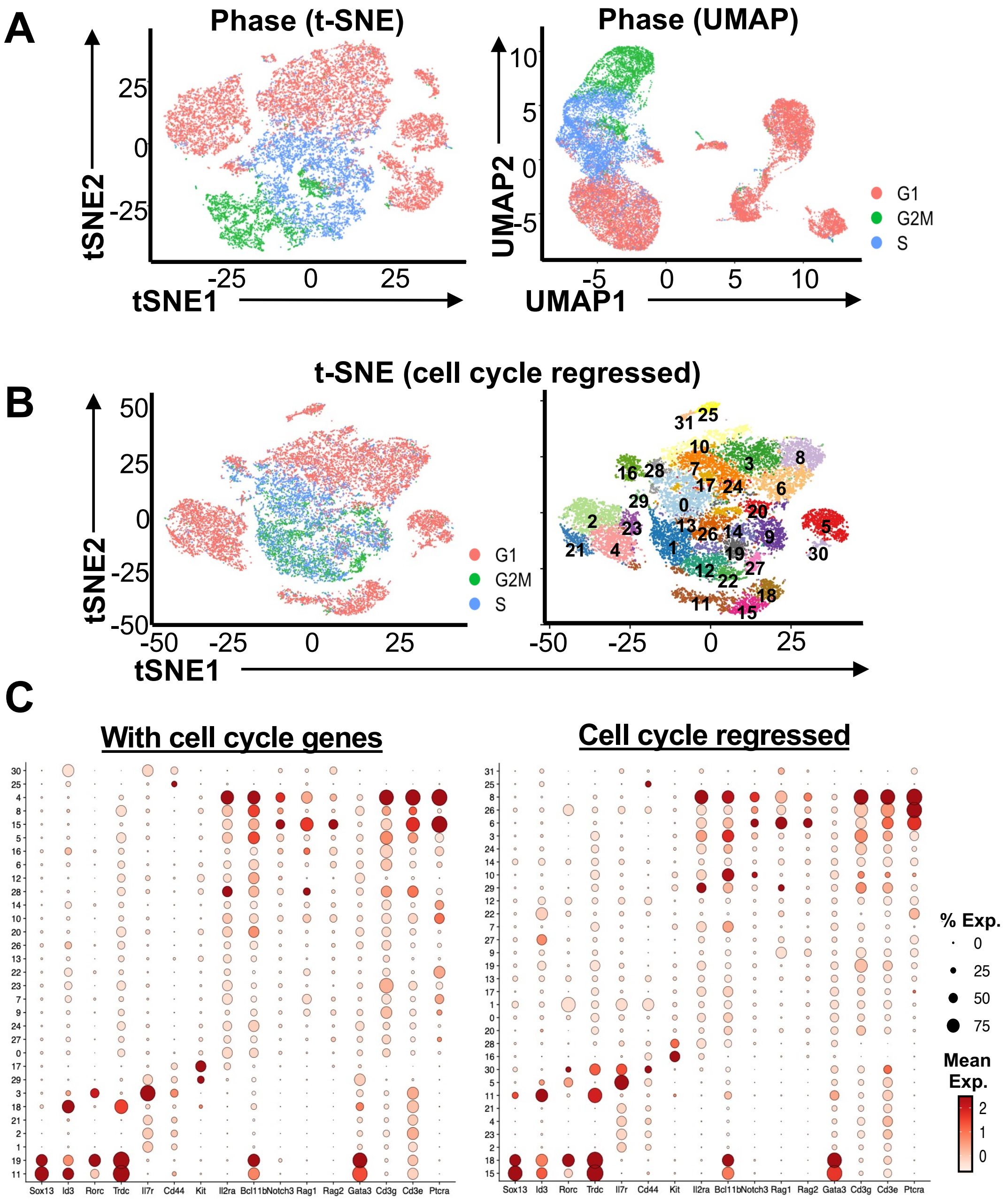

SUPPLEMENTARY FIGURE 3. Cell cycle has a minimal impact on the clustering of the DN thymocyte scRNA-seq data. **(A)** t-SNE (left) and UMAP (right) visualization of the integrated scRNA-seq data derived from the three 10X runs of DN and TCR$\gamma\delta^+$ thymocytes. Each cell was tagged as in G1, G2/M or S phase based on expression of cell cycle genes. **(B)** Cell cycle genes were first regressed out using Seurat's built-in regression model and clustered. The cells were then retagged to cell cycle stage (left). 31 distinct clusters were resolved (right). **(C)** Dot plot showing the expression of key markers genes across the clusters comparing the output with cell cycle genes left in or regressed out. Dot size indicates the percentage of cells within the cluster expressing the gene, while color saturation indicates average expression.

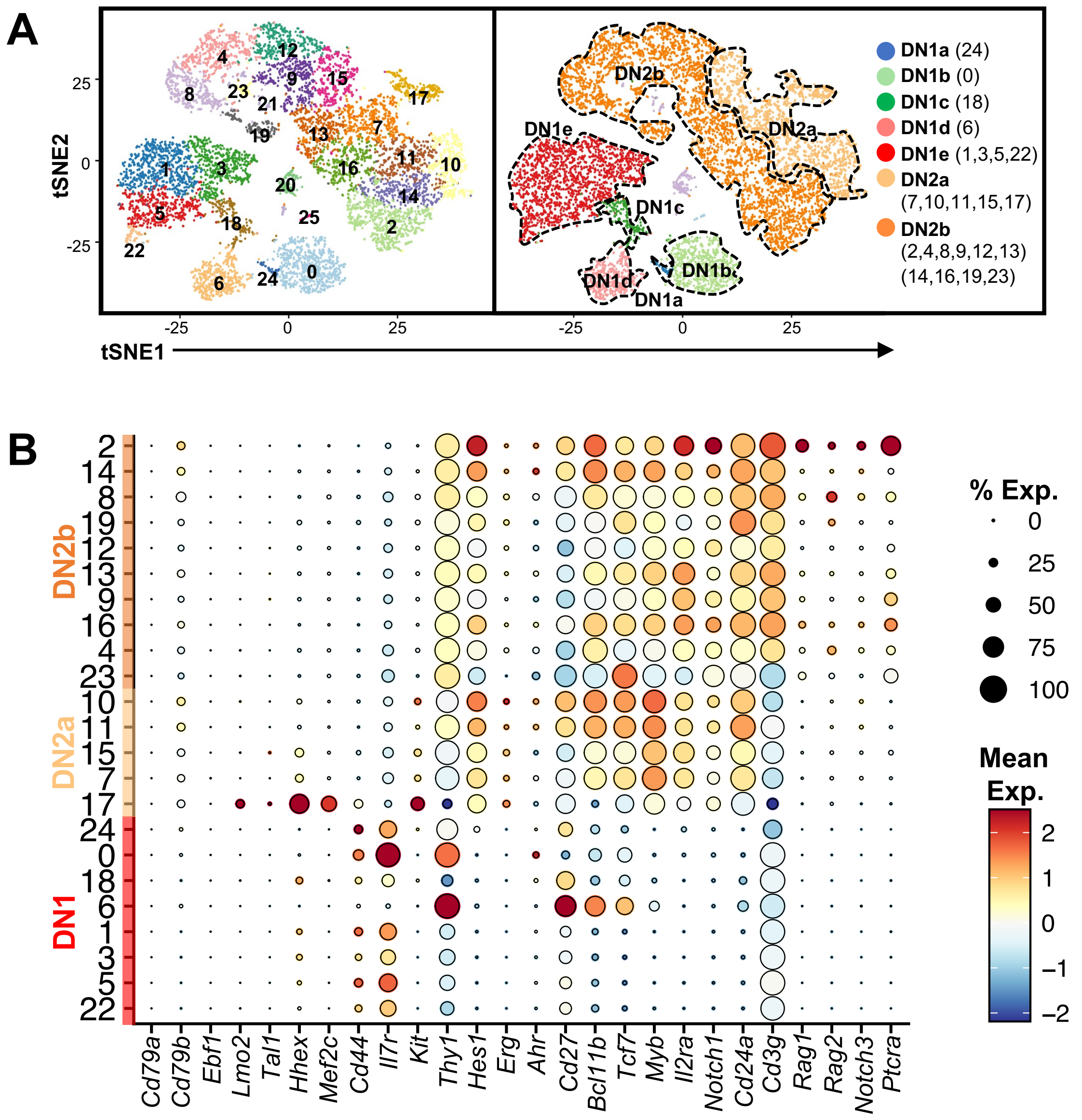

SUPPLEMENTARY FIGURE 4. Single-cell analysis of DN1 and DN2 thymocytes. **(A)** t-SNE projection of the data in Figure 4A. **(B)** Dot plot showing the expression of key markers genes previously shown to be differentially expressed between DN1, DN2a and DN2b. Cluster 20, 21 and 25 were identified as non-thymocytes (doublets and B cells) and were removed from downstream analyses.

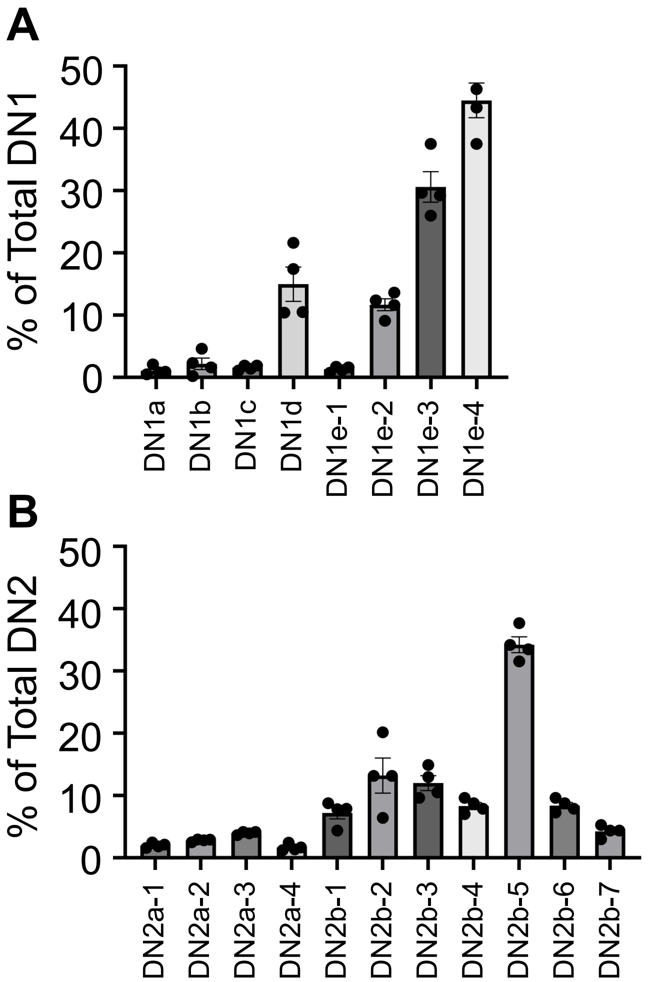

SUPPLEMENTARY FIGURE 5. Reproducible identification of the **(A)** eight DN1 and **(B)** 11 DN2 subpopulations by flow cytometry. Shown is the mean ± S.E.M., with individual experimental points.
